# Microvascular changes in the macular and parafoveal areas of multiple sclerosis patients without optic neuritis

**DOI:** 10.1038/s41598-022-17344-3

**Published:** 2022-08-03

**Authors:** Mihai Bostan, Jacqueline Chua, Yin Ci Sim, Bingyao Tan, Inna Bujor, Damon Wong, Gerhard Garhöfer, Cristina Tiu, Leopold Schmetterer, Alina Popa-Cherecheanu

**Affiliations:** 1 Carol Davila University of Medicine and Pharmacy, Bucharest, Romania; 2 Ophthalmology Emergency Hospital, Bucharest, Romania; 3 Singapore Eye Research Institute, Singapore National Eye Centre, 20 College Road, The Academia, Level 6, Discovery Tower, Singapore, 169856 Singapore; 4 Ophthalmology and Visual Sciences Academic Clinical Program, Duke-NUS Medical School, National University of Singapore, Singapore, Singapore; 5 SERI-NTU Advanced Ocular Engineering (STANCE), Singapore, Singapore; 6 School of Chemical and Biological Engineering, Nanyang Technological University, Singapore, Singapore; 7 Department of Clinical Pharmacology, Medical University Vienna, Vienna, Austria; 8 Department of Neurology, Emergency University Hospital, Bucharest, Romania; 9 Center for Medical Physics and Biomedical Engineering, Medical University Vienna, Vienna, Austria; 10 Institute of Molecular and Clinical Ophthalmology, Basel, Switzerland; 11 Department of Ophthalmology, Emergency University Hospital, 169 Splaiul Independentei, Level 8, District 5, 050098 Bucharest, Romania

**Keywords:** Diagnostic markers, Eye diseases, Neurological disorders, Neurology

## Abstract

Retinal imaging has been proposed as a biomarker for neurological diseases such as multiple sclerosis (MS). Recently, a technique for non-invasive assessment of the retinal microvasculature called optical coherence tomography angiography (OCTA) was introduced. We investigated retinal microvasculature alterations in participants with relapsing–remitting MS (RRMS) without history of optic neuritis (ON) and compared them to a healthy control group. The study was performed in a prospective, case–control design, including 58 participants (n = 100 eyes) with RRMS without ON and 78 age- and sex-matched control participants (n = 136 eyes). OCTA images of the superficial capillary plexus (SCP), deep capillary plexus (DCP) and choriocapillaris (CC) were obtained using a commercial OCTA system (Zeiss Cirrus HD-5000 Spectral-Domain OCT with AngioPlex OCTA, Carl Zeiss Meditec, Dublin, CA). The outcome variables were perfusion density (PD) and foveal avascular zone (FAZ) features (area and circularity) in both the SCP and DCP, and flow deficit in the CC. MS group had on average higher intraocular pressure (IOP) than controls (*P* < 0.001). After adjusting for confounders, MS participants showed significantly increased PD in SCP (*P* = 0.003) and decreased PD in DCP (*P* < 0.001) as compared to controls. A significant difference was still noted when large vessels (LV) in the SCP were removed from the PD calculation (*P* = 0.004). Deep FAZ was significantly larger (*P* = 0.005) and less circular (*P* < 0.001) in the eyes of MS participants compared to the control ones. Neither LV, PD or FAZ features in the SCP, nor flow deficits in the CC showed any statistically significant differences between the MS group and control group (*P* > 0.186). Our study indicates that there are microvascular changes in the macular parafoveal retina of RRMS patients without ON, showing increased PD in SCP and decreased PD in DCP. Further studies with a larger cohort of MS patients and MRI correlations are necessary to validate retinal microvascular changes as imaging biomarkers for diagnosis and screening of MS.

## Introduction

Multiple sclerosis (MS) is a chronic autoimmune disease of the central nervous system (CNS) in which inflammation, demyelination, and axonal loss occur even in the early stages of the disease^1^. It has been shown that autoreactive T-cells activated outside the CNS play an important role in the disease process. They cross the blood–brain barrier and are reactivated by local antigen-presenting cells^2^. Further, the secretion of proinflammatory cytokines stimulates microglial cells and astrocytes, recruits additional inflammatory cells, and triggers antibody production by plasma cells. This inflammatory process finally leads to tissue damage with the well described clinical consequences^2^. In addition to this well-understood immune mediated processes, vascular and metabolic factors are increasingly recognized to play a significant role in the pathophysiology of the disease^3^.

As such, there is increasing evidence that brain perfusion as measured using MRI is significantly affected in patients with MS^4,5^. However, the role of vascular changes in MS is complex and not yet fully understood. Optical coherence tomography angiography (OCTA) is a new diagnostic tool that allows for the in situ, high-resolution visualization of the individual vascular layers within the retinal capillary network^6^. OCTA visualizes the superficial, deep and choroidal vascular networks, and several algorithms have been proposed to extract quantitative data from OCT angiograms^6,7^. Given that the retina is a neural tissue and the eye is frequently affected by MS, OCTA may offer the unique opportunity to assess changes in brain vasculature by looking at vascular networks within the retina as a surrogate for the cerebral vasculature^8^. While there are a few OCTA studies investigating MS, there have been contradictory results^9^. While many studies reported a decrease in the vessel density (VD) in the superficial plexus in eyes without any history of optic neuritis (ON)^10–12^, one study reported a substantial increase in the VD in the superficial plexus only in MS patients without history of ON (MSNON) compared with controls^13^. Similarly, the findings of the deep plexus of eyes without any history of ON appeared contradictory: whereas one study reported a substantial decrease in VD^11^, another study observed an increase in VD^13^, yet other studies reported the deep vascular plexus was unaffected^14,15^. The reasons for these discrepancies are not clear, but may be related to differences in inclusion/exclusion criteria and/or analysis of OCTA data.

To address these gaps in knowledge, the purpose of the current study was to test whether there is a vascular rarefication in the microvasculature in MSNON compared to healthy, age- and sex-matched subjects. For this purpose, a new OCTA algorithm that accounts for potential measurement bias of foveal avascular zone (FAZ), OCT magnification correction with axial length measurements and projection artifacts of deep capillary plexus (DCP) was used.

## Methods

### Study design

The study followed a prospective, case–control design, which included patients with MS and healthy age- and sex-matched participants. The study complied with the tenets of the Declaration of Helsinki and was approved by the Emergency University Hospital Bucharest Institutional Ethics Committee (ID 11285). Written informed consent was obtained from all the participants before inclusion in the study.

### Study participants

To be included in the study each participant (with MS or healthy) had to be aged at least 18 years old, able to understand and willing to comply with the procedures and the actions as outlined in the study protocol. Any of the following excluded a participant (with MS or healthy) from the study: ocular surgery in the 3 months preceding the study, presence of ocular disease that would interfere with the study goals, such as glaucoma/-suspect/self-report glaucoma, diabetic retinopathy, retinal vein occlusion, choroidal neovascular membrane, age-related macular degeneration, sight-threatening ocular disease or any other clinically relevant eye disease. We also excluded the uncooperative patients with poor quality of images. All patients with MS had their diagnoses confirmed by their treating neurologists according to the 2017 McDonald criteria^16^. Patients were included if they had relapsing–remitting MS (RRMS), characterized by flare-ups of neurological symptoms with periods of remissions^17^, and were taking a disease-modifying treatment. Patients with a history of ON and/or patients with either primary progressive MS (PPMS) or secondary progressive MS (SPMS) were excluded from the study. All patients were referred to the Department of Ophthalmology in Bucharest for their regular ophthalmological eye check. Healthy control subjects were age- and sex-matched to the MS groups. Subjects with presence or history of a severe medical condition as judged by the investigators were excluded from the study.

### Ophthalmic examination

Each patient underwent a complete ophthalmic examination, including slit-lamp biomicroscopy and indirect funduscopy, visual acuity using the ETDRS acuity charts, OCTA scans of the macula, visual field testing to exclude any visual field defects, refraction, measurement of axial length of the eye (Carl Zeiss, IOL Master) to correct the OCT magnification effect, and applanation tonometry to measure intraocular pressure (IOP).

### Optical coherence tomography

All patients underwent an optic disc scan protocol (200 A-scans × 200B-scans: 6 × 6 mm) using the Cirrus spectral domain-OCT (Carl Zeiss Meditec, Inc, Dublin, CA, USA). The global peripapillary retinal nerve fiber layer (RNFL) thickness measurements were extracted from the optic disc OCT scans using the Cirrus Review Software (software version 11.0.0.29946).

### Optical coherence tomography angiography

All OCTA scans were performed by a single trained technician, using the Zeiss Cirrus HD-5000 Spectral-Domain OCT with AngioPlex OCTA (Carl Zeiss Meditec, Dublin, CA), that featured a central wavelength of 840 nm, a speed of 68,000 A-scan per second, an axial resolution of 5 µm and transverse resolution of 15 µm in tissue. The FastTrac motion correction, based on a line-scanning ophthalmoscope, was enabled to minimize motion artefacts during acquisition. Each participant received a 3 × 3 mm^2^ scan, with each scan consisting of an isotropic sampling (245 × 245 pixels) and four consecutive B-scans obtained at each raster location to compute the angiographic information using an optical microangiography protocol^18^.

A trained grader masked to the participant’s characteristics reviewed the quality of all OCTA scans. All B-scans were checked for alignment and segmentation errors. We excluded participants from the analysis if the OCTA images from both eyes were of poor quality (poor signal strength index < 6, significant motion artefacts visible as irregular vessel patterns on the *en face* angiogram, local weak signal caused by artefacts such as floaters, misalignment or incorrect segmentation)^19,20^. Both eyes of each participant were included in the analysis if the OCTA images from both eyes were of good quality.

Each scan was automatically segmented into the superficial capillary plexus (SCP) and DCP by a review software (Carl Zeiss Meditec, version 11.0.0.29946). The SCP spans the inner limiting membrane (ILM) to the inner plexiform layer (IPL), while the DCP spans the inner nuclear layer (INL) to the outer plexiform layer (OPL)^21^. Images were checked to ensure correct segmentation by the automated instrument software and no manual adjustment was needed. Projection artefacts from the overlying retinal circulation were removed from the DCP using the removal software that was integrated with the instrument. The choriocapillaris (CC) angiogram, spanning from 31 µm below the retinal pigment epithelium (RPE) to 40 µm below the RPE, was also extracted for analysis.

**Figure 1:**
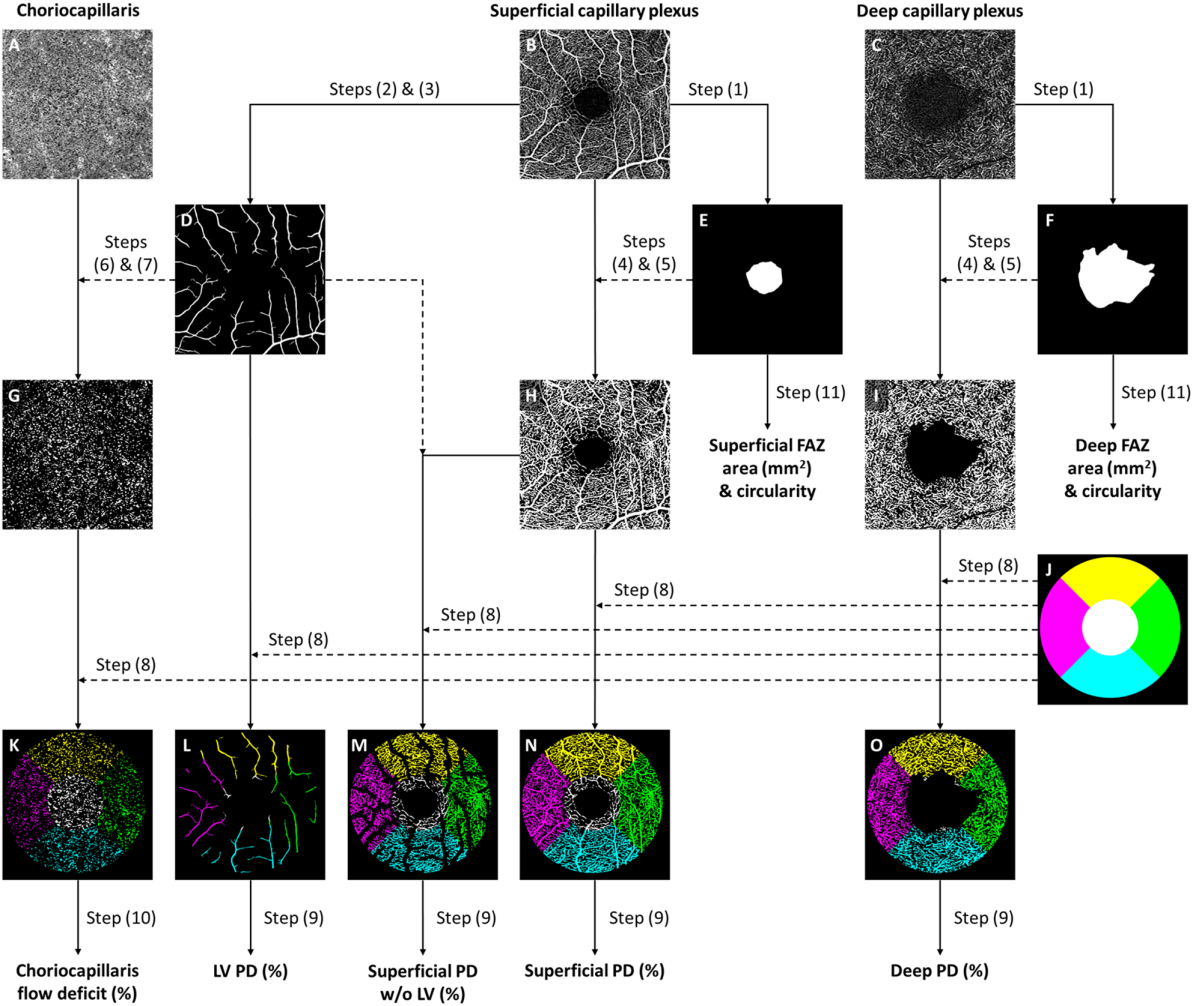
The framework of optical coherence tomography angiography (OCTA) image post-processing. (**A**–**C**) Raw OCTA images extracted from the OCTA machine. (**D**) Large vessels (LV) segmented and binarized from the superficial capillary plexus. (**E**,**F**) Foveal avascular zones (FAZ) manually delineated from the superficial and deep capillary plexuses. (**G**) Choriocapillaris flow voids binarized from the OCTA image. Large vessel artefacts were masked before binarization. (**H**,**I**) Vessels binarized from the superficial and deep capillary plexuses. FAZ regions were masked from the binarized images. (**J**) A magnification-corrected fovea-centered annulus mask with inner diameter of 1.0 mm and outer diameter of 2.5 mm. (**K**) Binarized choriocapillaris flow voids overlaid with annulus mask to perform regional quantification of flow deficit. (**L**–**O**) Binarized vascular images overlaid with annulus mask to perform regional quantification of perfusion density (PD).

OCTA images of the SCP, DCP and CC were subsequently loaded into a customized MATLAB (The MathWorks Inc., Natick, MA) algorithm. The framework of OCTA image processing involved the following steps (Fig. 1): (1) manually outlined the border of the FAZ of the superficial and deep vascular plexus angiograms^22^; (2) applied a combination of Gabor filter and Hessian-based filter to enhance the contrast of large vessels (LV) on the SCP, which is consequently (3) binarized^23,24^; (4) binarized the vessels on the SCP and DCP by setting a threshold at the mean intensity of the respective images, with subsequent (5) masking of FAZ regions; (6) removed large vessel artefacts from the CC angiogram, and (7) binarized the flow voids by setting a threshold that is 1 standard deviation below the mean intensity of the image; (8) performed region-based analysis with a fovea-centered annulus that has an inner diameter of 1.0 mm and outer diameter of 2.5 mm. Ocular magnification associated with OCTA scans was corrected at this step by rescaling the size of the annulus with Bennett’s formula^25^. The relationship between OCTA measurements and actual scan diameter was expressed by the formula ${s}_{actual}=p\times q\times s$, where ${s}_{actual}$ represents the actual scan length, $p$ represents the magnification factor for the camera of the imaging system, $q$ represents the magnification factor of the eye, further defined as $q=0.01306\times \left(axial length-1.82\right)$, and $s$ represents the scanning size of the OCTA protocol. A magnification-corrected annulus was hence determined for each scan by inserting axial length data into the formula. (9) Perfusion density (PD) of the vessels was computed as the percentage of vessel area per total imaged area in the annulus region of measurement. (10) Flow deficit in the CC was computed as the percentage of flow voids area per total imaged area in the annulus region of measurement. (11) FAZ area and circularity were computed for both the superficial and deep layer. FAZ circularity was computed as the ratio between the perimeter of the FAZ and the perimeter of an equivalent circle (i.e. circle of the same area). A value closer to 1 would hence indicate greater circularity.

### Statistical analysis

Shapiro–Wilk test was used to assess the normality of the distribution of the continuous variables. To compare the characteristics of participants between groups, Kruskal–Wallis test was performed for non-normally distributed continuous variables, independent t-test was performed for normally distributed continuous variables, and chi-square or Fisher's exact tests were performed for categorical variables. The effect of MS (independent variable) on OCTA variables (dependent variables) was assessed using multivariable linear regression analysis with generalized estimating equations (accounting for inter-eye correlation). In addition to age, gender, hypertension, and signal strength of OCTA scans, factors with *P* < 0.10 in the univariate model were included in the multivariate model^19,20^. The main outcome variables of the study were PD and FAZ features in both the SCP and DCP, and the secondary outcome was flow deficit in the CC. For either outcome, a *P* value < 0.05 was considered statistically significant. For characteristics of participants, a *P* value < 0.10 was considered statistically significant. Statistical analysis were performed using Stata version 16.0 (StataCorp LLC, College Station, TX).

### Ethics approval and consent to participate

The institutional review boards at all participating centers approved this study, and informed consent was obtained from the participants.

## Results

Of the 155 participants (*n* = 310 eyes) who were enrolled and imaged between August 2020 to January 2021, we excluded 74 eyes for poor scan quality, leaving 58 MS participants (*n* = 100 eyes) and 78 control participants (*n* = 136 eyes) with good quality OCTA for analysis. An overview of patients characteristics is given in Table 1. No significant difference in age and gender, the spherical equivalent, axial length, visual acuity measurements, or signal strength was observed between the groups. Of note, the MS group had on average a slightly higher IOP (healthy group: 15.4 ± 2.5 mmHg, MS group: 17.3 ± 2.8 mmHg; *p* < 0.001) and higher OCT scan quality (healthy group: 8.5 ± 1.0, MS group: 8.8 ± 0.9; *P* = **0.015**) than controls.

**Table 1:** Characteristics of participants by disease status.

Characteristics	Control (*n* = 78 patients; 136 eyes)	MS (*n* = 58 patients; 100 eyes)	*P* value*
Age	39 ± 11	41 ± 11	0.164
Gender, male / female	28 (36%) / 50 (64%)	19 (33%) / 39 (67%)	0.703
Spherical equivalent, D	− 0.09 ± 1.74	+ 0.19 ± 1.16	0.495
Axial length, mm	23.3 ± 0.1	23.2 ± 0.1	0.912
IOP, mmHg	15.4 ± 2.5	17.3 ± 2.8	**< 0.001**
Visual acuity, logMAR value (Snellen)	0.01 ± 0.06 (6/6^–1^)	0.01 ± 0.02 (6/6^–1^)	0.536
Visual field mean deviation (dB)	− 2.1 ± 2.5	–	–
Signal strength of OCT scans, out of 10	8.5 ± 1.0	8.8 ± 0.9	**0.015**
Signal strength of OCTA scans, out of 10	9.5 ± 0.9	9.5 ± 0.9	0.920

Data presented are mean (SD) or number (%), as appropriate.

*IOP* intraocular pressure, *OCT* optical coherence tomography, *OCTA* optical coherence tomography angiography.

Significant values are in bold.

*P value was obtained with Kruskal–Wallis test for non-normally distributed continuous variables, independent t-test for normally distributed continuous variables, and chi-square or Fisher’s exact tests for categorical variables.

In the univariate analysis, peripapillary RNFL thickness was significantly thinner in the MS compared to the control group (MS: 86.7 ± 1.6 μm, healthy: 95.3 ± 1.0 μm *P* < 0.001) whereas PD in the SCP was significantly higher in the MS compared to the control group (MS: 43.0 ± 0.3%, healthy: 41.9 ± 0.3% *p* = 0.007). This significant difference was still noted when the LV of the SCP were removed from the PD calculation (*P* = 0.014). In contrast, PD in the DCP was lower in the MS group (39.1 ± 0.6%) compared to the control group (41.6 ± 0.4%, *P* < 0.001).

Interestingly, FAZ at the deep layer was significantly larger (*P* = 0.005) and less circular (*P* < 0.001) in the eyes of MS participants than the control ones. FAZ at the superficial layer was also significantly less circular (*P* = 0.012) in the eyes of MS participants than the control ones. There was no statistically significant difference in LV PD, FAZ area in the SCP and flow deficit in the CC between the control group and MS group (*P* ≥ 0.185) (Table 2).

**Table 2:** Univariate analysis of optical coherence tomography angiography parameters with MS.

OCTA metrics	Control (*n* = 78 patients; 136 eyes)	MS (*n* = 58 patients; 100 eyes)	*P* value*
	Mean ± SE	Mean ± SE	
Peripapillary retinal nerve fiber layer thickness (μm)	95.3 ± 1.0	86.7 ± 1.6	**< 0.001**
**Perfusion density (%)**			
SCP	41.9 ± 0.3	43.0 ± 0.3	**0.007**
SCP w/o LV	28.7 ± 0.3	29.7 ± 0.3	**0.014**
LV	6.7 ± 0.1	6.6 ± 0.1	0.185
DCP	41.6 ± 0.4	39.1 ± 0.6	**< 0.001**
**Foveal avascular zone at superficial layer**			
Area (mm^2^)	0.25 ± 0.01	0.25 ± 0.01	0.857
Circularity	1.12 ± 0.01	1.17 ± 0.02	**0.012**
**Foveal avascular zone at deep layer**			
Area (mm^2^)	1.06 ± 0.03	1.24 ± 0.06	**0.005**
Circularity	1.17 ± 0.01	1.28 ± 0.02	**< 0.001**
**Flow deficit (%)**			
CC	16.4 ± 0.1	16.6 ± 0.2	0.270

*CC* choriocapillaris, *DCP* deep capillary plexus*, LV* large vessels, *OCTA* optical coherence tomography angiography, *SCP* superficial capillary plexus, *SE* standard error.

Significant values are in bold.

Peripapillary RNFL thickness remained significantly thinner in the MS as compared to the control group in the multivariate analysis. After adjusting for age, gender, hypertension, signal strength of OCTA scans and IOP, PD in the SCP of the MS and control groups were 43.1 ± 0.3% and 41.9 ± 0.3%, respectively (*p* = 0.003). A significant difference was still noted when the LV of the SCP was removed from the PD calculation (*P* = 0.004). Notably, PD in the DCP of the MS and control groups was significantly different (39.2 ± 0.6% and 41.5 ± 0.3%, respectively; *P* < 0.001). Similar to the univariate analysis, FAZ at the DCP remained significantly larger (*P* = 0.005) and less circular (*P* < 0.001) in the eyes of MS participants than the control ones. However, FAZ at the SCP was no longer less circular (*P* = 0.071) in the eyes of MS participants compared to the controls.

**Figure 2:**
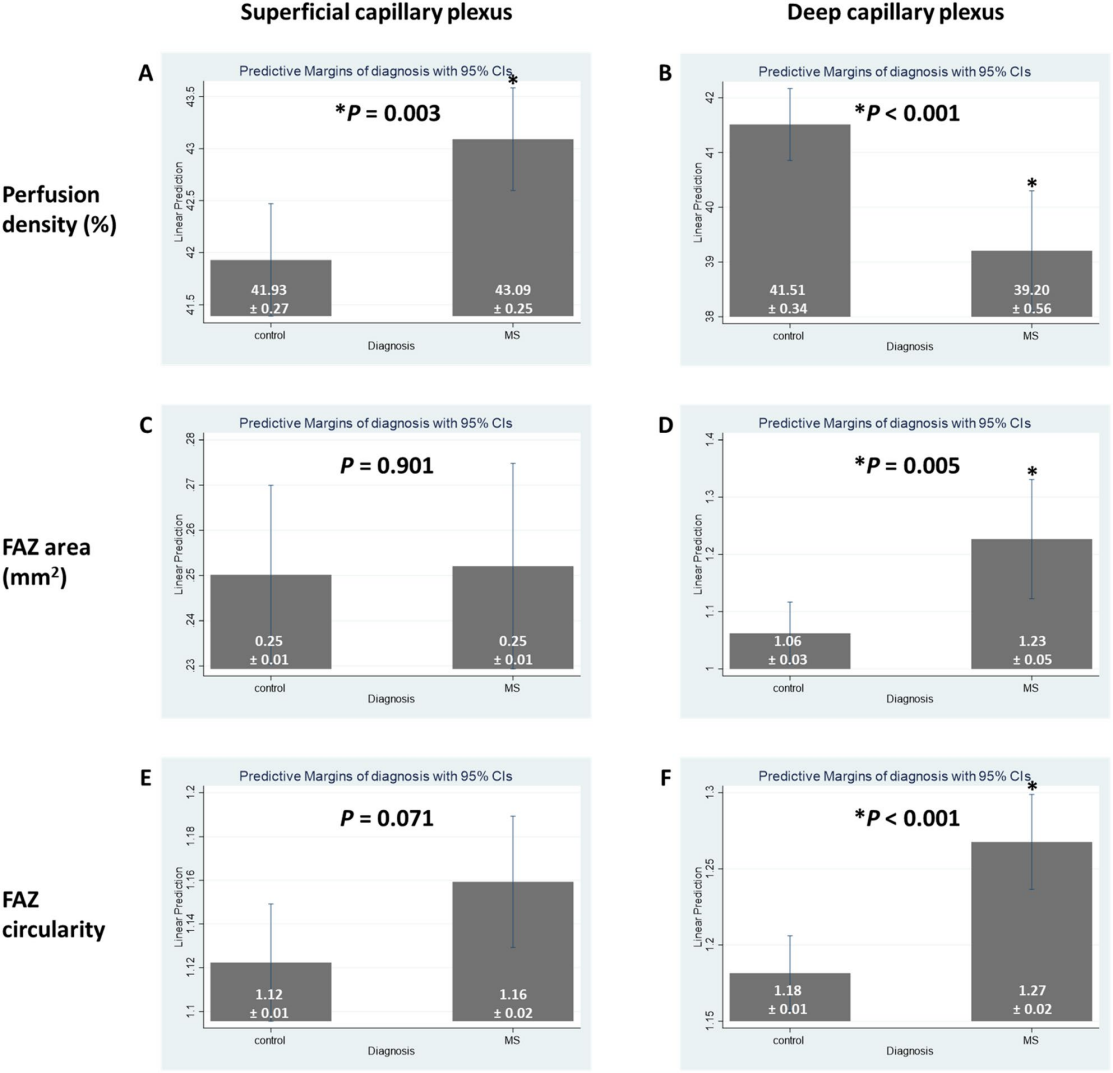
Distribution of (**A**) superficial perfusion density, (**B**) deep perfusion density, (**C**) superficial foveal avascular zone (FAZ) area, (**D**) deep FAZ area, (**E**) superficial FAZ circularity, and (**F**) deep FAZ circularity, stratified by participants with MS and controls. Data and *P* values shown are after adjustment for age, gender, hypertension, signal strength of optical coherence tomography angiography (OCTA) scans, and intraocular pressure. The asterisk symbol (*) indicates a statistical significance of *P* < 0.05 when compared to the controls.

**Figure 3:**
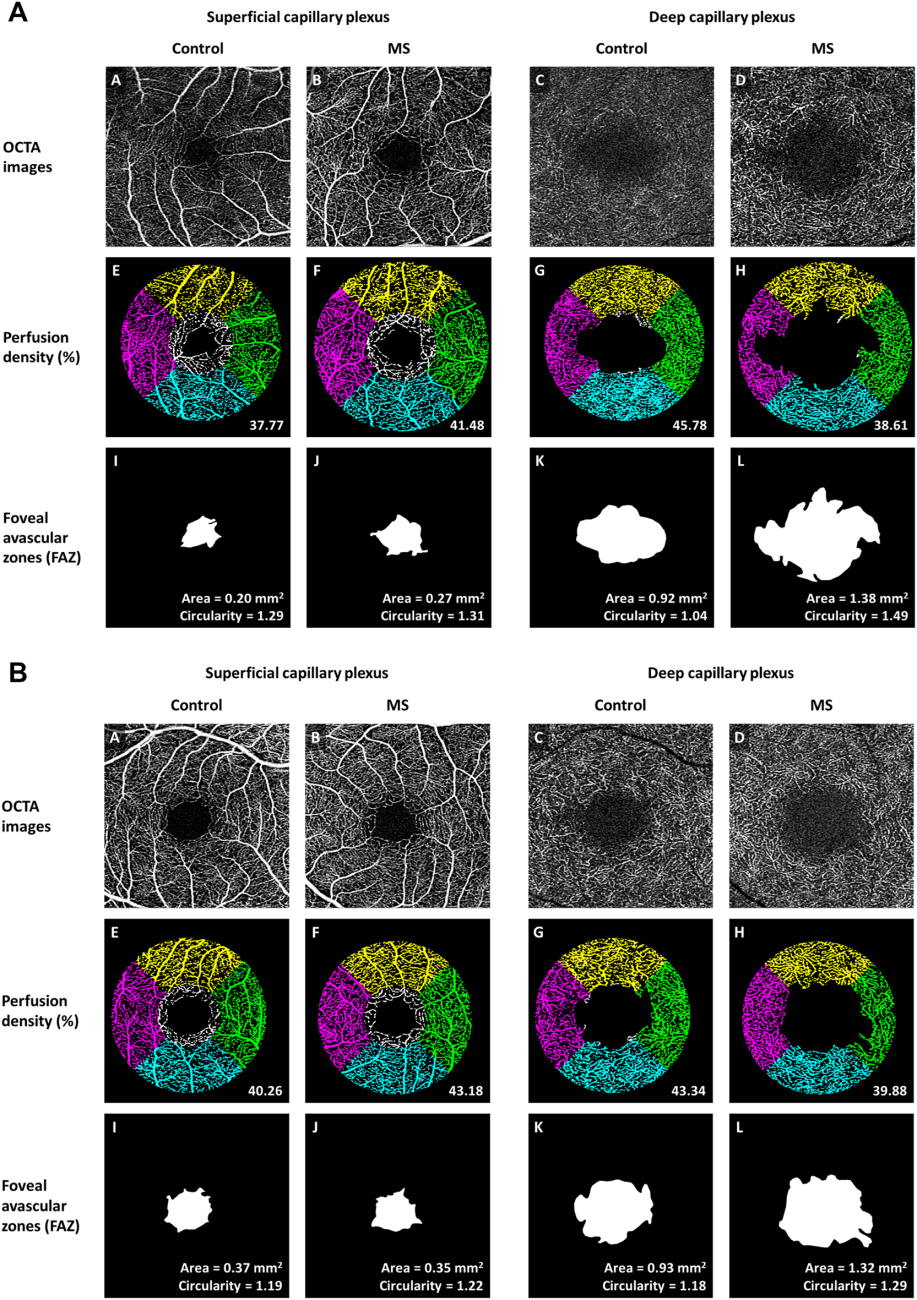
(**A**) Optical coherence tomography angiography (OCTA) images of the superficial capillary plexus (SCP; A,B) and deep capillary plexus (DCP; C,D) extracted from the OCTA machine. (E–H) Perfusion density (PD) maps of the macular annulus region, showing retinal vasculature in controls (E,G) and MS participants (F,H). MS participants showed increased PD in the SCP and decreased PD in the DCP compared to controls. (I-L) Foveal avascular zones (FAZ) delineated from the SCP and DCP, showing FAZ features in controls (I,K) and MS participants (J,L). MS participants showed larger and less circular FAZ than controls only in the DCP, and not in the SCP. (**B**) Optical coherence tomography angiography (OCTA) images of the superficial capillary plexus (SCP; A,B) and deep capillary plexus (DCP; C,D) extracted from the OCTA machine. (E,H) Perfusion density (PD) maps of the macular annulus region, showing retinal vasculature in controls (E,G) and MS participants (F,H). MS participants showed increased PD in the SCP and decreased PD in the DCP compared to controls. (I–L) Foveal avascular zones (FAZ) delineated from the SCP and DCP, showing FAZ features in controls (I,K) and MS participants (J,L). MS participants showed larger and less circular FAZ than controls only in the DCP, and not in the SCP.

There was no statistically significant difference in LV PD, FAZ area in the SCP and flow deficit in the CC between the control group and MS group (*P* ≥ 0.186) (Table 3). Figure 2 and Fig. 3 further illustrate the graphical representation and OCTA images of PD and FAZ features in the SCP and DCP of the control and MS groups, respectively. We did not find any significant associations between OCTA parameters and duration of MS (Table 4) or number of episodes (Table 5).

**Table 3:** Multivariate analysis of optical coherence tomography angiography parameters with MS.

OCTA metrics	Control (*n* = 78 patients; 136 eyes)	MS (*n* = 58 patients; 100 eyes)	*P* value*
	Mean ± SE	Mean ± SE	
Peripapillary retinal nerve fiber layer thickness (μm)	95.4 ± 1.0	86.6 ± 1.6	**< 0.001**
**Perfusion density (%)**			
SCP	41.9 ± 0.3	43.1 ± 0.3	**0.003**
SCP w/o LV	28.7 ± 0.3	29.8 ± 0.3	**0.004**
LV	6.70 ± 0.07	6.55 ± 0.08	0.186
DCP	41.5 ± 0.3	39.2 ± 0.6	**< 0.001**
**Foveal avascular zone at superficial layer**			
Area (mm^2^)	0.25 ± 0.01	0.25 ± 0.01	0.901
Circularity	1.12 ± 0.01	1.16 ± 0.02	0.071
**Foveal avascular zone at deep layer**			
Area (mm^2^)	1.06 ± 0.03	1.23 ± 0.05	**0.005**
Circularity	1.18 ± 0.01	1.27 ± 0.02	**< 0.001**
**Flow deficit (%)**			
Choriocapillaris	16.4 ± 0.1	16.6 ± 0.2	0.215

*DCP* deep capillary plexus*, LV* large vessels, *OCTA* optical coherence tomography angiography, *SCP* superficial capillary plexus, *SE* standard error.

Significant values are in bold.

*Adjusted for age, gender, hypertension, signal strength of OCT or OCTA scans, and intraocular pressure.

**Table 4:** Univariate analysis of optical coherence tomography angiography parameters with duration of MS.

OCTA metrics	β	95% CI	*P* value*
**Perfusion density (%)**			
SCP	− 0.02	− 0.09 to 0.05	0.559
SCP w/o LV	− 0.03	− 0.13 to 0.07	0.558
LV	0.01	− 0.03 to 0.05	0.641
DCP	− 0.13	− 0.30 to 0.03	0.113
**Foveal avascular zone at superficial layer**			
Area (mm^2^)	− 0.01	− 0.01 to 0.01	0.758
Circularity	0.01	− 0.01 to 0.01	0.693
**Foveal avascular zone at deep layer**			
Area (mm^2^)	0.01	− 0.01 to 0.02	0.316
Circularity	0.01	− 0.01 to 0.01	0.288
**Flow deficit (%)**			
Choriocapillaris	− 0.01	− 0.05 to 0.03	0.796

*CI* confidence interval, *DCP* deep capillary plexus*, LV* large vessels, *OCTA* optical coherence tomography angiography, *SCP* superficial capillary plexus.

**Table 5:** Univariate analysis of optical coherence tomography angiography parameters with number of MS episodes.

OCTA metrics	β	95% CI	*P* value*
**Perfusion density (%)**			
SCP	0.04	− 0.06 to 0.14	0.449
SCP w/o LV	0.05	− 0.05 to 0.15	0.304
LV	− 0.01	− 0.03 to 0.02	0.760
DCP	0.02	− 0.10 to 0.15	0.743
**Foveal avascular zone at superficial layer**			
Area (mm^2^)	0.01	− 0.01 to 0.01	0.717
Circularity	− 0.01	− 0.01 to 0.01	0.663
**Foveal avascular zone at deep layer**			
Area (mm^2^)	0.01	− 0.01 to 0.01	0.964
Circularity	− 0.01	− 0.01 to 0.01	0.390
**Flow deficit (%)**			
Choriocapillaris	− 0.01	− 0.05 to 0.03	0.717

*CI* confidence interval, *DCP* deep capillary plexus*, LV* large vessels, *OCTA* optical coherence tomography angiography, *SCP* superficial capillary plexus.

## Discussion

The aim of the current study was to evaluate retinal microvascular changes, specifically at the capillary network level, in patients with MSNON. Our study shows that after accounting for the potential measurement bias of FAZ, OCT magnification correction with axial length measurements and projection artifacts of DCP, RRMS participants showed significantly increased PD in SCP, and significantly decreased PD in DCP as compared to healthy controls. Our data support the concept that there are alterations in the retinal microvasculature in RRMS that can be detected by means of OCTA, which may potentially be used as imaging biomarkers to identify and screen for MS.

In the past, OCT has been used to investigate potential neurodegenerative changes in retina. There is compelling evidence that patients with MS show thinning of the retinal neural tissue, indicating that changes in neurodegenerative changes of patients with MS are reflected in the eye. In particular, a recent meta-analysis of more than 25,000 patient records shows pronounced retinal thinning mostly in the peripapillary retinal nerve fiber layer as well as in the macular ganglion cell layer and IPL^26^.

Although there is a general agreement that retinal thinning is consistently observed in patients with MS, OCTA studies investigating the microcirculation in patients with MS have revealed contradictory results^9^. As such, it has been reported that SCP vascularization is increased^13^ and combined RNFL + GCIPL is decreased^13^, whereas other studies found decreased^8,10,27^ vascular density and decreased^28^ or the same^8^ RNFL in patients with MS. This also holds true for other OCTA derived parameters such as DCP, where the increased^13^ and decreased values^11,27^ have been observed, with retinal thinning association^11,13^. In our recent OCT paper on the same cohort of patients, we demonstrated thinning of the peripapillary RNFL and inner macular ganglion cell complex^29^. Importantly, the paper highlighted that the capability of OCT in MS differentiation is made more robust by accounting OCT scans for individual anatomical differences and incorporating information from both optic disc and macular regions, representing markers of axonal damage and neuronal injury, respectively.

Our results are in accordance with one previous study^13^, which reported increased VD of the SCP and decreased RNFL + GCIPL, but they are in contrast to other, more recent experimental data, where most of the other studies revealed a decreased VD^8,10–12^ or did not report any differences^30^ for MSNON, with RNFL decreased^8,11,12^ or uninvestigated^10^.

Only a few OCTA studies have investigated DCP VD in MS individuals, but again there is a lack of agreement between studies. One study^13^ showed a significant increase in VD in MSNON, whereas some data indicate^11^ a significant reduction in VD, and others^8,12,15,30^ did not observe any differences.

The reason for the contradicting results in both SCP and DCP has not yet been elucidated but may be due to methodological and technical limitations of OCTA and selection of study population. Study populations were heterogeneous in the above mentioned studies: some included only patients with RRMS, whereas others included all clinical forms of MS. Further, some studies included only MSNON, whereas others studied MS patients with history of ON (MSON) as well as MSNON.

Further, technical limitations may account for the differences. Accurate assessment of OCTA metrics is challenging and may be affected by the physiologic variability of FAZ^31–33^ and projection artifacts^31,34,35^. This holds particularly true for the FAZ in the DCP. However, in the past only a few OCTA studies accounted for the FAZ in the deep plexus^11,30^. This is a major limitation because the deep FAZ is considerably larger than superficial FAZ^31,36^. In addition, while some studies have removed the projection artifacts in the DCP^11–13^, others did not. In the current study, we quantified the PD of the DCP without the influence of FAZ and projection artifacts, which reduced measurement bias.

In the current study, superficial FAZ was significantly less circular in the eyes of MS participants than controls in univariate analysis, an effect that was not observed anymore in the multivariate analysis. We did not find any differences in superficial FAZ area between groups, neither in the univariate, nor in multivariate analysis. Interestingly, deep FAZ was significantly larger and less circular in MS participants than controls in both, univariate and multivariate analysis, which is compatible with the decreased PD of the DCP.

Overall, the FAZ features are limited by the physiological variability and may not be such a robust biomarker for the diagnosis and screening of MS^22,31,37–40^.

The reason for the observed increase of VD in the SCP is unclear. One can speculate that it is related to diffuse chronic inflammation and consequently to increased angiogenesis^41–43^. Inflammation may be regarded as causative to vascular changes in MS^15^. Widespread and subtle inflammation in MS has been observed in histopathologic studies^13^, in both the retina^44^ and the brain^45^. The increased VD in SCP could also reflect a compensatory response to retinal tissue hypoperfusion^46^ linked to angiogenesis. Tissue hypoperfusion can affect tissue oxygenation and induce hypoxia-like changes including vasodilatation in the retina^47^. Such hypoxia can be the trigger for Vascular Endothelial Growth Factor (VEGF) release and several other pro-angiogenic molecules, which can lead to increased VD^13^. Further studies are needed to elucidate this issue.

The current study has some strength and limitations that warrant further discussion. First we included a well phenotype cohort of RRMS individuals who were diagnosed according to the 2017 McDonald criteria^16^, a standardized study methodology which has been used in a variety of cohorts^24,31,48–51^. Second, we have a high signal strength among our participants, which confirms the validity of our results. Third, we adjusted the possible confounding effects of age and gender during multivariate analysis and excluded possible confounding factors, such as PPMS, SPMS and any clinically relevant eye disease. Also, we excluded patients with any history of ON, in order to avoid a bias related to optic nerve direct damage, because ON can cause a decrease in VD in the posterior pole of the eye^52^, independently of the presence of MS.

However, our present study also has a few limitations. First, a quarter of eyes examinations were excluded because of poor quality OCTA scans. Such high exclusion is comparable to other studies using OCTA^12^ since the quality of the OCTA scans is dependent on good patient compliance and excellent target fixation. In the future, higher scan speeds with eye motion correction in challenging situations will eliminate this limitation^31,53^. Second, cerebral perfusion was not measured in the same study cohort, and the link between the eye and the brain in tissue perfusion could not be established. Third, it remains unclear whether the changes of retinal capillaries are related to the prediction of disability progression, whole brain atrophy or the differential neuroprotective effects of disease-modifying therapies. Finally, an increase in OCTA metric has been reported in patients with cataracts in the 3-month follow-up period after cataract surgery^54^. Although our inclusion criterion was to allow individuals with more than 3 months of ocular surgery to participate, none of the included participant have undergone prior ocular surgery. As such, the OCTA metric was not confounded by ocular surgery.

## Conclusion

Our study shows that there are microvascular changes in the parafoveal retina of RRMS patients without ON, showing increased PD in SCP and decreased PD in DCP. Thus, our results confirm that OCTA may be useful to detect even subclinical changes. However, further studies with a larger cohort of MS patients and MRI correlations are necessary to validate retinal microvascular changes as imaging biomarkers for diagnosis and screening of MS.

## Data Availability

The datasets used and/or analyzed during the current study are available from the corresponding authors on reasonable request. The dataset(s) supporting the conclusions of this article is(are) included within the article.

## Abbreviations

CC: Choriocapillaris
CI: Confidence interval
CNS: Central nervous system
DCP: Deep capillary plexus
FAZ: Foveal avascular zone
ILM: Inner limiting membrane
INL: Inner nuclear layer
IOP: Intraocular pressure
IPL: Inner plexiform layer
LV: Large vessels
MS: Multiple sclerosis
MSNON: MS patients without history of optic neuritis
MSON: MS patients with history of optic neuritis
n: Number
OCTA: Optical coherence tomography angiography
ON: Optic neuritis
OPL: Outer plexiform layer
PD: Perfusion density
PPMS: Primary progressive MS
RPE: Retinal pigment epithelium
RRMS: Relapsing–remitting MS
SCP: Superficial capillary plexus
SD: Standard deviation
SE: Standard error
SPMS: Secondary progressive MS
VD: Vessel density
